# The Book World of Medicine and Science

**Published:** 1895-01-05

**Authors:** 


### THE BOOK WORLD OF MEDICINE AND SCIENCE.

DEFECTS IN PLUMBING AND DRAINAGE WORK. Described by FRANCIS VACHER, M.D.

This practical and useful little book is, for the most part, a republication of a series of notes contributed to the *Health Journal* during a series of years, partly the result of the author's own observations, partly of information furnished by correspondents in various localities.

Beginning with defects in connection with pipes for carrying off rain-water from roofs, the author deals successively with yard and area drains, sink waste-pipes, bath and lavatory waste pipes and overflows, water-closet fittings, soil and ventilating pipes, house drains, and domestic cisterns and hot and cold water apparatus. Each example is illustrated by diagram, which clearly explains the defect it refers to. It would have made reference from text to diagram much easier had the two been in all cases on the same page; and a little more attention to scale would also have been helpful.

In connection with the subject of roof drainage, or, as the author calls it, "rain conducting," the evils arising from cracked pipes, defective joints, and the consequent soakage of wet into or around walls are pointed out. An omission which might with advantage be rectified in a future edition is the neglect to point out the importance of fixing rain-water pipes clear of the walls to which they are attached. The reason for this is that when a pipe cracks either at its socket or elsewhere the water which escapes will run down the pipe and not down the brickwork.

Some remarkable and instructive examples of evil-doing in the matter of sink wastes are given, notably Fig. 24, which shows a sink waste-pipe connected to a 6 in. drain, into which at the same point a hopper w.c. basin discharged, the latter being unprovided with any means of flushing. Another example in the chapter on bath and lavatory waste shows a bath waste-pipe discharging into the basin of a closet. This, the author tells us, was an arrangement "carried out by a competent plumber at his own house." Let us hope he is not, or at any rate was not, a registered plumber.

In dealing with water-closet fittings the author wisely avoids recommending any particular maker's wares; but the diagram of a suitable form of apparatus which he gives is badly drawn and defective in several points. For example, it has no flushing rim, and as drawn it could not be successfully cast in one piece of earthenware. The most remarkable example of bad workmanship in this section is that of a hopper basin discharging into a pail; the contents of the latter overflowed and found their way (?) into the open mouth of a drain some little distance away. Next to th is which the author justly calls abominable closet, was the kitchen, and the foul air and stinks had free access thereto through a wall, the joints of which were almost destitute of mortar, and the open joints of the boarded floor.

Some curious examples of ventilating soil pipes are given. Of one the author says that it was put up with the approval of the local surveyor and medical officer. It consists of a 4 in. lead pipe brought through the wall and bent down into an iron pipe. At the bend of the lead pipe a hole had been drilled, and an iron pipe half-an-inch in diameter had been fixed above, but just not long enough to connect with the lead pipe. The soil pipe, therefore, ventilated itself through the hole at the bend into the adjacent window.

In the last chapter some useful examples are given o defective plumbing in connection with cisterns and hot and cold water service. The common mistake of not protecting expansion pipes from frost is illustrated by an example in which a copper cylinder was completely wrecked through this cause.

The book is a very valuable addition to the ever-increasing stock of sanitary literature, and deserves to be widely read.

